# Can a semi-quantitative method replace the current quantitative method for the annual screening of microalbuminuria in patients with diabetes? Diagnostic accuracy and cost-saving analysis considering the potential health burden

**DOI:** 10.1371/journal.pone.0227694

**Published:** 2020-01-21

**Authors:** Yaerim Kim, Seokwoo Park, Myung-Hee Kim, Sang Hoon Song, Won Mok Lee, Hye Soon Kim, Kyubok Jin, Seungyeup Han, Yong Chul Kim, Seung Seok Han, Hajeong Lee, Jung Pyo Lee, Kwon Wook Joo, Chun Soo Lim, Yon Su Kim, Dong Ki Kim

**Affiliations:** 1 Division of Nephrology, Department of Internal Medicine, Keimyung University School of Medicine, Daegu, Korea; 2 Division of Nephrology, Department of Internal Medicine, Seoul National University Hospital, Seoul, Korea; 3 Department of Internal Medicine, Seoul National University College of Medicine, Seoul, Korea; 4 Department of Biomedical Sciences, Seoul National University College of Medicine, Seoul, Korea; 5 Department of Dental Hygiene, College of Health Science, Eulji University, Gyeonggi-do, Korea; 6 Department of Laboratory Medicine, Seoul National University Hospital, Seoul, Korea; 7 Department of Laboratory Medicine, Keimyung University School of Medicine, Daegu, Korea; 8 Division of Endocrinology and Metabolism, Department of Internal Medicine, Keimyung University School of Medicine, Daegu, Korea; 9 Kidney Research Institute, Seoul National University College of Medicine, Seoul, Korea; 10 Division of Nephrology, Department of Internal Medicine, Seoul National University Boramae Medical Center, Seoul, Korea; International University of Health and Welfare, School of Medicine, JAPAN

## Abstract

**Objectives:**

Diabetes is a global epidemic, and the high cost of annually and quantitatively measuring urine albumin excretion using the turbidimetric immunoassay is challenging. We aimed to determine whether a semi-quantitative urinary albumin-creatinine ratio test could be used as a screening tool for microalbuminuria in diabetic patients.

**Methods:**

We assessed the diagnostic accuracy of the semi-quantitative method. The costs of false results in the semi-quantitative method were calculated based on the annual probability of disease progression analyzed through a systematic literature review and meta-analysis. The pooled long-term cost-saving effect of the semi-quantitative method compared with the quantitative test was assessed using a Markov model simulating a long-term clinical setting. Diagnostic accuracy and the cost-saving effect were also validated in an independent external cohort.

**Results:**

Compared with the quantitative test, the semi-quantitative method had sensitivities of 93.5% and 81.3% and specificities of 61.4% and 63.1% in the overall sample of diabetic patients (n = 1,881) and in diabetic patients with eGFR ≥60 ml/min/1.73 m^2^ and a negative dipstick test (n = 1,110), respectively. After adjusting for direct and indirect medical costs, including the risk of disease progression, which was adjusted by the meta-analyzed hazard ratio for clinical outcomes, it was determined that using the semi-quantitative method could save 439.4 USD per person for 10 years. Even after adjusting the result to the external validation cohort, 339.6 USD could be saved for one diabetic patient for 10 years.

**Conclusions:**

The semi-quantitative method could be an appropriate screening tool for albuminuria in diabetic patients. Moreover, it can minimize the testing time and inconvenience and significantly reduce national health costs.

## Introduction

Diabetic nephropathy (DN) is the most common cause of chronic kidney disease (CKD), and the risk for end-stage renal disease (ESRD) is approximately 12-fold higher in diabetic patients than in non-diabetic patients.[1–4] Guidelines have recommended measuring the urine albumin-creatinine ratio (uACR) and estimated glomerular filtration rate (eGFR) annually in diabetic patients.[5, 6]

Quantitative measurement of the uACR with an immunoturbidimetric assay is the standard method, with excellent precision for determining the 24-hour urine albumin excretion level. However, this method is expensive when used as a screening tool for detecting microalbuminuria and requires specialized equipment, trained personnel, and a considerable amount of time. Additionally, it requires the specimen to be transported to a central laboratory because the assay cannot be performed in primary clinics where most diabetic patients receive medical care. To counter these drawbacks, a semi-quantitative uACR test has been developed as a point-of-care testing (PoCT) method to screen for microalbuminuria. The strengths of PoCT include faster decisions, improved adherence to treatment, and reduced incidence rates of complications.[7] Recently, researchers have focused on the economic advantage of PoCT. However, different results have been obtained, with a lack of evidence regarding the cost-saving effect of PoCT.[8–10] To consider the opportunity cost of false negatives and false positives, a cost-saving analysis needs to be performed to investigate whether the semi-quantitative method could replace the standard method for a disease that affects a large proportion of the population. Therefore, we focused on the diagnostic accuracy of the semi-quantitative method for detecting microalbuminuria and the cost-saving effect for diabetic patients, calculating the opportunity cost of false results via a meta-analysis.

## Materials and methods

### Development and validation cohorts

The study was performed in two independent cohorts. For the development cohort, diabetic patients diagnosed as per American Diabetes Association guidelines who visited the Seoul National University Hospital between June 2017 and January 2018 were initially screened in the study.[11] Before screening for microalbuminuria, patients with microscopic hematuria, pyuria, or urinary casts were excluded. All clinical and laboratory data were retrospectively examined by reviewing the electronic health records. A subset with an eGFR ≥60 ml/min/1.73 m^2^ and negative dipstick test for proteinuria was selected to assess the diagnostic accuracy of the semi-quantitative method for detecting microalbuminuria in diabetic patients without clinically evident DN.

In the validation cohort, we performed an additional analysis using diabetic patients who had their uACRs checked at the Keimyung University Dongsan Hospital between July 2019 and September 2019. The exclusion criteria were applied the same as those for the development cohort, all data were retrospectively reviewed using electronic health records.This study was approved by the local Institutional Review Board of Seoul National University Hospital (D-1704-018-843) and Keimyung University Dongsan Hospital (DSMC 2019-08-016) and registered at ClinicalTrials.gov (NCT03238547).

### Sample collection and Laboratory analysis

Freshly voided random urine samples were obtained and stored for a maximum of 24 hours at 4 ^o^C. All urine samples were analyzed within 6 hours of collection using the following three methods: dipstick urinalysis, quantitative testing, and the semi-quantitative method. Dipstick urinalysis was performed in the biomedical examination room using a SYSMEX UC-3500 analyzer (Sysmex Co., Ltd., Kobe, Japan). Urine albumin was quantitatively measured using the immunoturbidimetry method with a TBA 120FR analyzer (Toshiba Medical Systems Corp., Tokyo, Japan). The urinary creatinine level was analyzed by the Jaffe kinetic reaction on Ci16000 and TBA 120FR analyzers (Toshiba Medical Systems Corp., Tokyo, Japan). The coefficients of variance were 5.6% and 3.6% for urine albumin and creatinine, respectively.

Serum creatinine (sCr) was measured using the alkaline picrate Jaffe kinetic method by a Hitachi 7600 analyzer (Toshiba Medical Systems Corp., Tokyo, Japan), and the assay was calibrated to standardized creatinine measured using the IDMS via corrected equations: calibrated sCr = 1.00 × measured sCr– 0.3 (mg/dL). The estimated GFR was calculated based on the Chronic Kidney Disease Epidemiology Collaboration equation.

In the validation cohort, all of the methods for sample preparation were the same as those for the development cohort. The types of equipment differed as follows: Roche cobas U6500 analyzer (Fritz Hoffmann-La Roche, Basel, Switzerland) for dipstick urinalysis, STRATEC SR 300 analyzer (Beckman Coulter Corp., California, United States) for urine albumin, Selectra ProS analyzer (ELITech Group, Puteaux, France) for urine creatinine, and Roche-Hitachi 8000 cobas C702 analyzer (Fritz Hoffmann-La Roche, Basel, Switzerland) for serum creatinine. Urine albumin and was analyzed with the radio-immunoanalytical (RIA) method with an albumin RIA kit (Beckman Coulter Corp., California, United States). The method for analyzing urine and serum creatinine was the same as that in the development cohort.

### Semi-quantitative test for uACR

The process for testing the semi-quantitative method was similar to the dipstick test. Urine albumin and creatinine were separately analyzed with the URiSCAN^®^ 2ACR system (YD-Diagnostics Co., Yongin, Korea) after placing the stick on the read device following brief exposure to the urine. Urine albumin was assayed using an immunoturbidity method with the Autokit Micro Albumin analyzer (FUJIFILM Wako Diagnostics, Osaka, Japan). Analysis of the urine creatinine level was performed by enzymatic assay with the L-Type Creatinine M analyzer (FUJIFILM Wako Diagnostics, Osaka, Japan). We performed the tests at 3 separate experimental sites with 3 different operators using the URiSCAN^®^ 2ACR strip and URiSCAN^®^ Pro urine analyzer (YD–Diagnostics Co.) to confirm the within-day precision. Between-day precision was analyzed with 10 separate measurements per day for 10 days and was represented by the reproducibility based on the Clinical and Laboratory Standards Institute guideline EP5–A2.[12] We conducted precision analysis with 3 different categories of uACR, namely, <30, 30–300, and ≥300 mg/g. Moreover, the sensitivity analysis and diagnostic accuracy test were performed based on dichotomous results, with a cutoff value of 30 mg/g.

### Modeling for cost-saving analysis and cost assessment

#### Model construction

**Fig 1:**
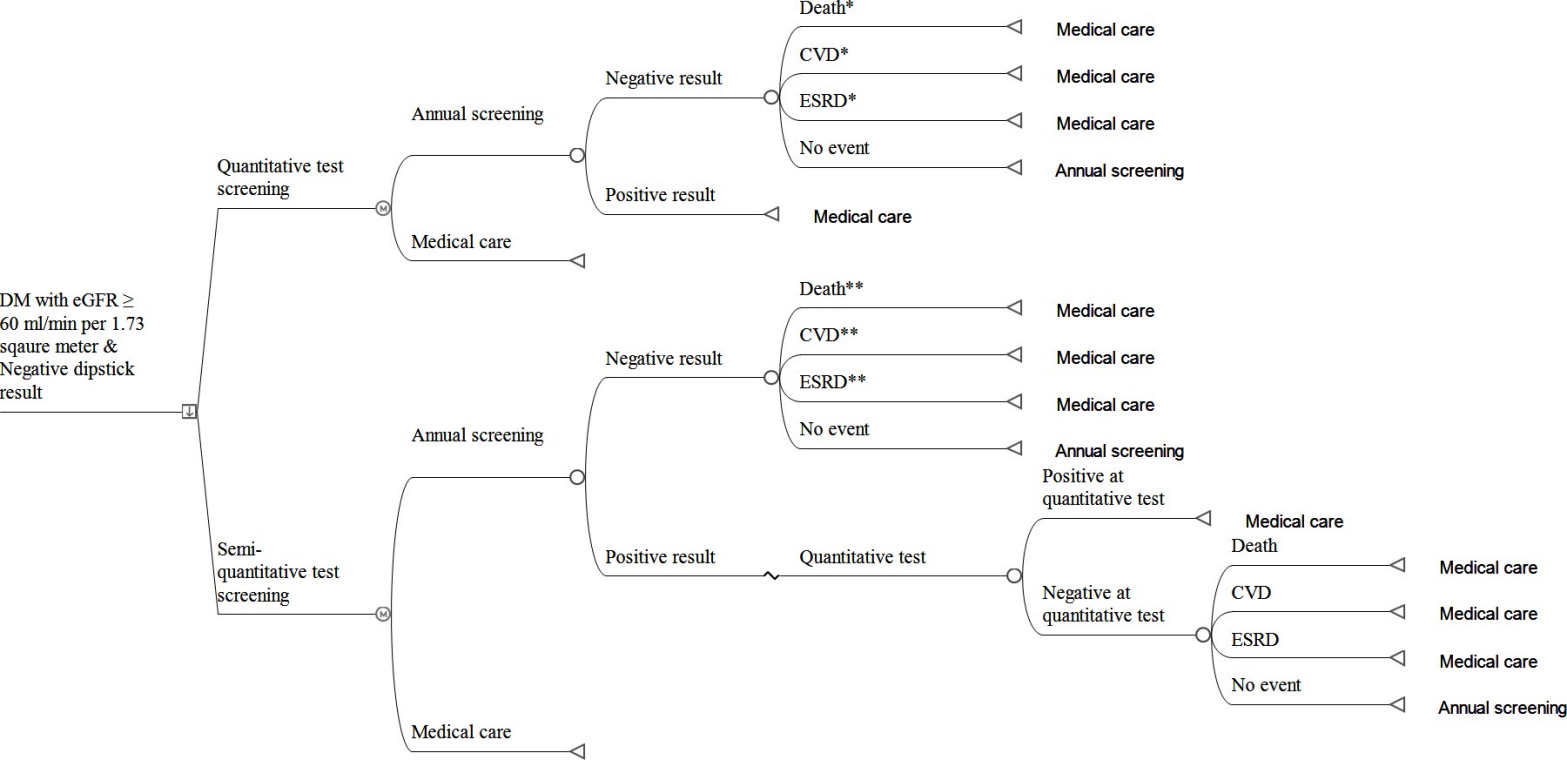
Structure of the Markov model. The square denotes a decision node where the quantitative or semi-quantitative screening strategy can be chosen. The circles represent chance nodes in which subsequent events occur at probabilities allocated to each node. The letter M in a circle indicates a Markov node. The triangles represent terminal nodes, where subjects return to the Markov node from which they started the cycle and start the next cycle again. Descriptions accompanying terminal nodes designate the path for the subjects to follow at the next Markov cycle. * Annual probabilities of these clinical events were derived from diabetic patients with normo-albuminuria. ** Putatively, both true negative and false negative fractions are present in the SemiQ negative population. The occurrences from both cases were included. For the population with false negatives, the hazard ratio for clinical outcomes was assessed in micro- or overt albuminuric diabetic patients. Additionally, for the population with true negative results, the hazard ratio for clinical outcomes was assessed in normo-albuminuric diabetic patients. Abbreviations: CVD, cardiovascular disease; DM, diabetes mellitus; ESRD, end-stage renal disease.

We conducted cost-saving analyses using a Markov model simulating a long-term clinical setting. The model is illustrated in Fig 1. Screening tests were performed annually; hence, the cycle length was 1 year, and the time horizon was 10 years. Those testing positive on the quantitative test (Q positive) were assumed to have micro- or overt albuminuria and thus stopped undergoing screening. Even if participants were normo-albuminuric on a quantitative test (Q negative), they were still susceptible to clinical outcomes (i.e., ESRD, cardiovascular disease [CVD], or death) to some extent. Those remaining without clinical outcomes were subject to the next the Markov cycle. For the semi-quantitative strategy, test positivity (SemiQ positive) was confirmed by the quantitative test. Depending on the confirmatory test results, for the false positive population, we assessed the probabilities of clinical outcomes in normo-albuminuria, while the true positive population, which had micro- or overt albuminuria, proceeded to medical care and were terminated from Markov cycle. Those testing negative on the semi-quantitative test (SemiQ negative) had the possibility of developing clinical events. We considered the possibility that a population with true negative results could progress to non-albuminuric outcomes and the population with false negative results could progress to albuminuric outcomes. Remainders without clinical events entered the next cycle.

#### Input parameters

Parameter values are described in Table 1. The probabilities of positive or negative test results at annual screening were derived from the study results that we performed (Table 2). Regarding the SemiQ-positive or SemiQ-negative population, the true or false result rate of confirmatory quantitative tests was also obtained from the results of our study. Incidences of clinical outcomes, referred from previous reports performed in diabetic patients with normo-albuminuria,[13, 14] were converted to probabilities by the formula: probability = 1-e^{-(rate×time)}.[15] For cases with micro- or overt albuminuria, incidences of clinical outcomes were estimated by multiplying the meta-analyzed hazard ratios to incidences in normo-albuminuric cases.

**Table 1:** Baseline input values used for cost-saving analyses.

Parameter	Baseline value	Reference
Direct medical cost for screening ($)		
Quantitative method	17.76	
Semi-quantitative method	0.86	
Direct nonmedical cost ($)		
Transportation	9.265	
Indirect medical costs for healthcare ($)		
End-stage renal disease	13,149.62	HIRA annual report
Cardiovascular disease	25,634.48	HIRA annual report
All-cause mortality	6,810.38	HIRA annual report
Incidences of clinical outcomes, normo-albuminuria (per year)		
End-stage renal disease	0.0154	Ref 14
Cardiovascular disease	0.0095	Ref 14
All-cause mortality	0.0006	Ref 13
Relative hazards of clinical outcomes, albuminuria versus normo-albuminuria*		
End-stage renal disease	3.432 (95% CI, 2.757–4.271)	Fig 2
Cardiovascular disease	1.315 (95% CI, 1.250–1.384)	Fig 2
All-cause mortality	1.480 (95% CI, 1.408–1.556)	Fig 2
Probabilities (distribution)		
Negative at quantitative screening	Beta (917, 193)**	Table 2
Negative at semi-quantitative screening	Beta (615, 495)**	Table 2
False negative among the negative population at SemiQ screening	Beta (36, 579)**	Table 2
True positive at Q test confirmation among the positive population at SemiQ test screening	Beta (157, 338)**	Table 2

* These values were used to estimate incidences of clinical outcomes among participants with albuminuria.

** Beta distribution.

**Table 2:** The concordance of urine albumin/creatinine ratio using the semi-quantitative method and quantitative testing.

	Quantitative testing (mg/g)	semi-quantitative method			Sensitivity	Specificity	*Over detected fraction	**Under detected fraction
		<30	30–300	≥300				
All diabetes (n = 1,881)	<30	664 (61.4)	408 (37.7)	9 (0.8)	93.5	61.4	22.2	2.8
	30–300	52 (10.8)	350 (72.9)	78 (16.2)				
	≥300	0 (0.0)	17 (5.3)	303 (94.7)				
Diabetes with eGFR ≥60 ml/min/m^2^ and dipstick (-) (n = 1,110)	<30	579 (62.9)	333 (36.3)	5 (0.5)	81.3	63.1	30.5	3.2
	30–300	36 (19.4)	142 (76.3)	8 (4.3)				
	≥300	0 (0.0)	2 (28.6)	5 (71.4)				

*, number of false positives/total number of population x 100

**, number of false negatives/total number of population x 100

eGFR, estimated glomerular filtration rate

#### Cost estimation

We calculated the pooled cost difference according to the measured differences between the semi-quantitative and quantitative methods. We included the direct medical costs for receiving the semi-quantitative or quantitative tests, direct nonmedical costs for transportation, and indirect costs for disease progression or premature death. To include strategy-dependent differences in health outcomes in the analyses, we focused on unavoided clinical outcomes that occurred in participants declared negative by the screening tests. Medical expenses for these events were added to the total cost of each strategy to comprehensively compare the economic values of the screening methods. Additional expenses for the quantitative assay and transportation were included for patients who were positive according to the semi-quantitative method.

The incidence rates of the clinical outcomes in normo-albuminuric diabetic patients were the previously reported values of 0.95%, 1.54%, and 0.06% for death,[13] CVD,[14] and ESRD,[14] respectively. The baseline annual medical costs were 6,810.4 USD per person per year (PPPY) for ESRD and 13,149.6 USD PPPY for CVD based on the Health Insurance Review and Assessment (HIRA) data mentioned above.[16] The expected cost of death was 25,634.5 USD PPPY.[16] The discount rate for both costs and health outcomes were considered as 3% per year.[17] The pooled cost-saving effect was assessed by comparing the cost of the semi-quantitative method with that of the quantitative test.

#### Sensitivity analysis

We performed one-way sensitivity analyses to address model uncertainties. Changes in the amount of cost savings by the semi-quantitative method were evaluated, while input parameters varied. The costs of screening tests, transportation, and clinical outcomes were varied by 20% from the baseline values. Incidences of clinical outcomes, which were used to calculate annual probabilities, were also varied by 20%. Hazard ratios (HRs) for clinical outcomes were varied within the range of 95% confidence intervals.

### Meta-analysis for probabilities of major clinical outcomes

The probabilities of major clinical outcomes occurring before the next annual screening were evaluated with a systematic literature review and subsequent meta-analysis. This review was performed following the Preferred Reporting Items for Systematic Reviews and Meta-analysis statement (S1 File). All included studies met the following inclusion criteria for the analysis: (1) patients with a confirmed diagnosis of diabetes; (2) studies considered renal function in terms of serum eGFR and proteinuria; (3) study population consisting of diabetic patients with normal renal function; (4) study outcome limited to all-cause mortality, CVD, and ESRD; and (5) manuscript written in English. Exclusion criteria were as follows: (1) studies considering interventions such as medication or lifestyle modification; (2) research for identifying the accuracy of a diagnostic tool; and (3) studies involving molecular analyses or biomarkers. We used the terms “mortality” or “myocardial infarction” or “cardiovascular disease” or “stroke” or “cerebrovascular disorder” or “end-stage renal disease” combined with “diabetes mellitus” and “albuminuria” for selecting eligible studies from the PubMed, MEDLINE, and EMBASE databases. Two different authors independently searched articles according to the above criteria published in web-based databases from January 1970 to January 2018. Finally, we found the articles that were maximally fitted to our requirement for calculating the relative HRs. We obtained the HRs and 95% confidence intervals (CIs) of major clinical outcomes in diabetic patients with albuminuria and eGFR ≥60 ml/min/1.73 m^2^ compared to those of normo-albuminuric diabetic patients.

### Health burden of false results: Financial resources and statistical information

Medical expenses were defined by the total cost of the treatment, which was determined to be valid by the Korea HIRA review process. Each medical expense is the sum of the amounts paid by the patient and the National Health Insurance Service. We obtained information on annual medical costs from the National Health Insurance Statistical Annual Report 2016 from the HIRA. Medical diseases were classified by the International Classification of Diseases version 10 in the report, and we extracted data using the codes I 20–25, I 50, I 60–63, and N 17–19. Estimates of the cost of death were based on research by the National Evidence-based Healthcare Collaborating Agency, which measured the value of the extension of life in Korea for one year by using the maximum payment intention as the willingness to pay.[18, 19] The cost of transportation was deduced from the HIRA report in 2005, and we reflected the price index from 2005 to 2016 in the inflation rate of 28.7%.[20] Statistical data regarding the population and the mortality rate according to age were extracted from the Korean Statistical Information Service. The prevalence of diabetes was multiplied by the reported incidence of diabetes among the population older than 20 years in Korea.

### Statistical analysis

To compare the baseline characteristics between the overall diabetes group and the study group, we used the Mann-Whitney U test for continuous variables and the chi-square test for categorical variables. Sensitivity and specificity were analyzed using the chi-square test and were calculated based on the cutoff vales of ≥30 mg/g for positive results and by <30 mg/g for negative results for the uACR.

In the cost-saving analysis, all cost values were presented in USD based on the exchange rate of the year in which the medical expenses were incurred (annual average exchange rate, 2016; 1 USD = 1,160 KRW). Meta-regression analyses were performed using the meta package with the metagen function in *R* software to estimate the pooled effect sizes between patients with albuminuria and patients without albuminuria for clinical outcomes such as ESRD, CVD, and death. Heterogeneity between studies was quantified using *I*_2_ statistics. Heterogeneity was considered statistically significant when P was <0.05, and the hazard ratio from the random effects model was used to estimate the cost for each health outcome. Data were analyzed with R software version 3.5.1 (Comprehensive R Archive Network: http://cran.rproject.org) and TreeAge Pro version 2019 (TreeAge Software, Inc.). In all analyses, P <0.05 was considered statistically significant.

## Results

### Development and validation cohorts

In the development cohorts, among 1,881 diabetes patients, 1,369 patients had an eGFR ≥60 ml/min/1.73m^2^. Among these patients, we finally selected 1,110 with normal results in the urinary dipstick test. There were 917 (82.6%) patients with uACR <30 mg/g, 186 (16.8%) with 30 to 300 mg/g, and 7 (0.6%) with ≥300 mg/g (S1 Fig).

Compared with all patients, the group of patients with eGFR ≥60 mL/min/1.73m^2^ and negative dipstick results was younger (median age 64.0 [IQR 14.0] vs. 66.0 [IQR 15.0] years). Most participants had a normal range of uACR <30 mg/g, and the group with eGFR ≥60 ml/min/1.73m^2^ and negative dipstick results had a higher rate than that of all diabetic patients (82.6% vs 57.5%) (S1 Table).

In the validation cohort, there were 431 diabetic patients. Among them, 301 patients showed an eGFR ≥60 ml/min/1.73m^2^ and normal urinary dipstick test results. The proportion with micro (14.3%) or overt-albuminuria (1.0%) was similar that in the development cohort. The baseline characteristics are described in S2 Table.

### Diagnostic accuracy of the semi-quantitative uACR method

The concordance between the semi-quantitative and quantitative methods was highest in the ≥300 mg/g uACR category in the overall diabetes group (94.7%). The sensitivity and specificity were 93.5% (748/800) and 61.4% (664/1,081), respectively (Table 2). The under-detected fraction was 2.8% (52/1,881) in the overall diabetic group. This means that out of 100 screened participants with diabetes, 42.5 had a uACR over 30 mg/g, and 39.7 of them would be correctly classified, while 2.8 of them would remain undetected by the semi-quantitative method. For the patients with eGFRs ≥60 ml/min/1.73 m^2^ and negative dipstick results, the sensitivity and specificity were 81.3% (157/193) and 63.1% (579/917), respectively. The under-detected fraction accounted for 3.2% (36/1,110) of the patients, meaning that out of 100 screened diabetic participants with normal renal function and negative dipstick results, only 17.4 had a uACR over 30 mg/g, and 3.2 of them would receive false negative results on the semi-quantitative test.

The number needed for screening was lower for the semi-quantitative method than for the dipstick test in both the overall diabetes group and in the group of diabetic patients with normal renal function, indicating that the semi-quantitative method is more efficacious than the dipstick method as a screening tool for albuminuria in diabetic patients (Table 3).

**Table 3:** Efficacy of the semi-quantitative method for albuminuria screening in patients with diabetes.

	Number	Number needed to screen	True positive	False negative	False positive
Semi-quantitative method					
All	1,881	1.1	93.5	6.5	38.6
Dipstick negative	1,303	1.2	83.3	16.7	37.4
eGFR ≥60 ml/min/1.73 m^2^	1,369	1.1	90.2	9.8	38.2
eGFR ≥60 ml/min/1.73 m^2^ and Dipstick negative	1,110	1.2	81.3	18.7	36.9
Dipstick method					
All	1,881	1.5	65.6	34.5	5.0
eGFR ≥60 ml/min/1.73m^2^	1,369	1.9	52.7	47.3	4.6

eGFR, estimated glomerular filtration rate

The within-day precision was expressed by linearity, with values of 0.853, 0.814, and 0.811 for urine albumin and 0.931, 0.929, and 0.930 for urine creatinine at three different sites (S2 Fig). The coefficient of variance was <10% and showed 100% agreement in reflectance photometry with the quantitative method according to different ranges and sites (S3 Table).

### Cost-saving analysis based on the meta-analysis of the relative risks of clinical outcomes

**Fig 2:**
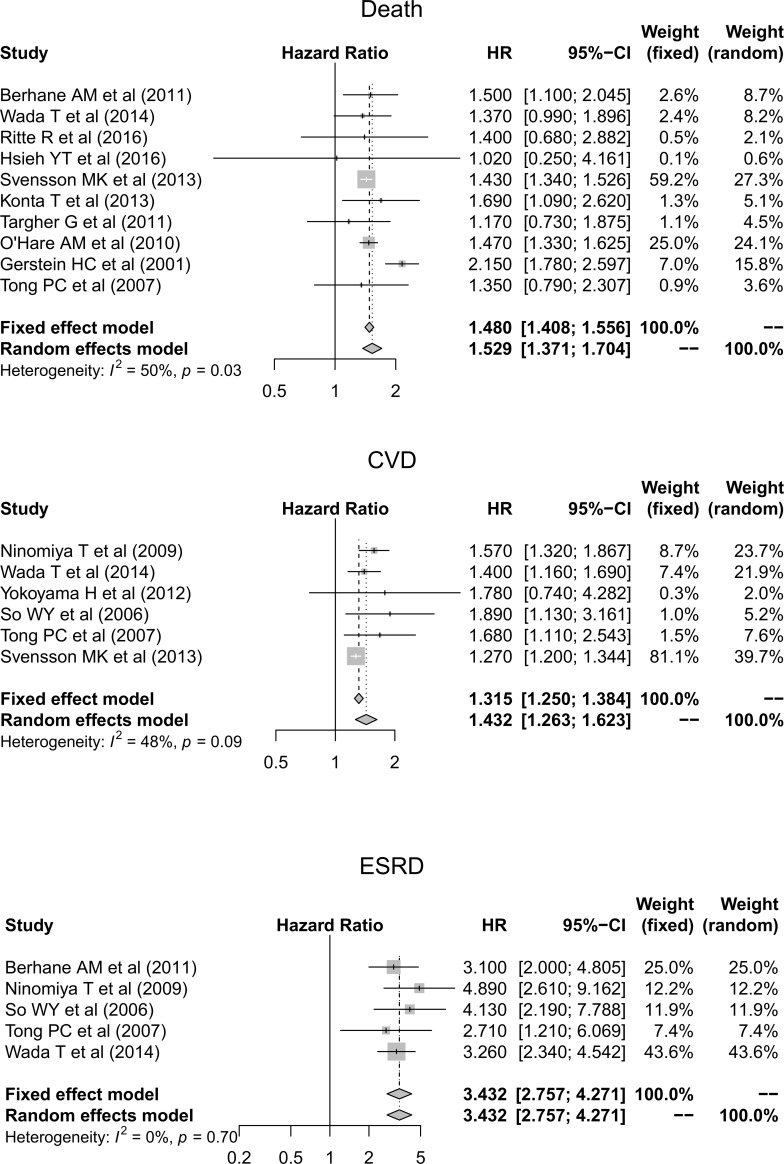
Relative hazard ratios for major clinical outcomes such as death, cardiovascular disease, and end-stage renal disease in diabetic patients with albuminuria and eGFR ≥60 ml/min/1.73 m^2^ compared to normo-albuminuric diabetic patients. Abbreviations: CVD, cardiovascular disease; CI, confidence interval; ESRD, end-stage renal disease; HR, hazard ratio.

To identify the induced costs associated with false results, the relative HRs for clinical outcomes were evaluated based on the results of a systematic review. In total, 7,085 articles were initially identified. After filtering by title and abstract and excluding duplicates, we reviewed the detailed contents of 45 articles, and finally, 13 articles were deemed eligible for inclusion (S3 Fig). Among these articles, 9 included results regarding all-cause mortality, 6 included results regarding CVD, and 5 included results regarding ESRD. We next meta-analyzed the different HRs in diabetic patients with and without albuminuria. The pooled HRs were 1.48 (95% CI, 1.41–1.56), 1.31 (95% CI, 1.25–1.38), and 3.27 (95% CI, 2.58–4.13) for all-cause mortality, CVD, and ESRD, respectively (Fig 2).

We considered all fractions of true and false results in the semi-quantitative test compared to the quantitative test, as described in Table 3. Based on the pooled HRs of the clinical outcomes, we adjusted the medical cost for each fraction of the results. The overall calculated costs for the direct and indirect medical expenses are illustrated in Table 4. Screening by the semi-quantitative method reduced the total cost for 10 years in one diabetic patient by 23.8% (439.4 USD) even after considering all disease progression probabilities in the false negative fraction. Moreover, after adjusting for the prevalence of diabetes, 200 billion USD could be saved nationwide.

**Table 4:** Cost-saving analysis of semi-quantitative compared with quantitative methods of a 10-year screening strategy for individuals over 20 years old in the total Korean population.

	Quantitative strategy (per person)	Semi-quantitative strategy (per person)
Initial screening for urine albumin to creatinine ratio, $*	80.72	4.38
Transportation, $	84.22	47.15
*Total costs for screening*, *$*	*164*.*95*	*51*.*53*
Confirmatory quantitative test for the positive fraction from the semi-quantitative strategy, $		
Quantitative test	-	40.31
Transportation	-	21.03
Cost of the under detected fraction, $ (95% CI)		
End-stage renal disease	15.34	13.16
Cardiovascular disease	754.57	576.99
All-cause mortality	910.11	702.52
*Summed costs (crude)*, *$*	*1844*.*97*	*1405*.*54*
*Summed costs (discounted)*, *$*	*1701*.*25*	*1286*.*36*
*Saved cost (crude)*, *$*		*439*.*44*
*Saved cost (crude)*, *%*		*23*.*8*

*1 USD = 1,160 KRW (Annual average exchange rate, 2016). Baseline year of cost, 2016.

**Fig 3:**
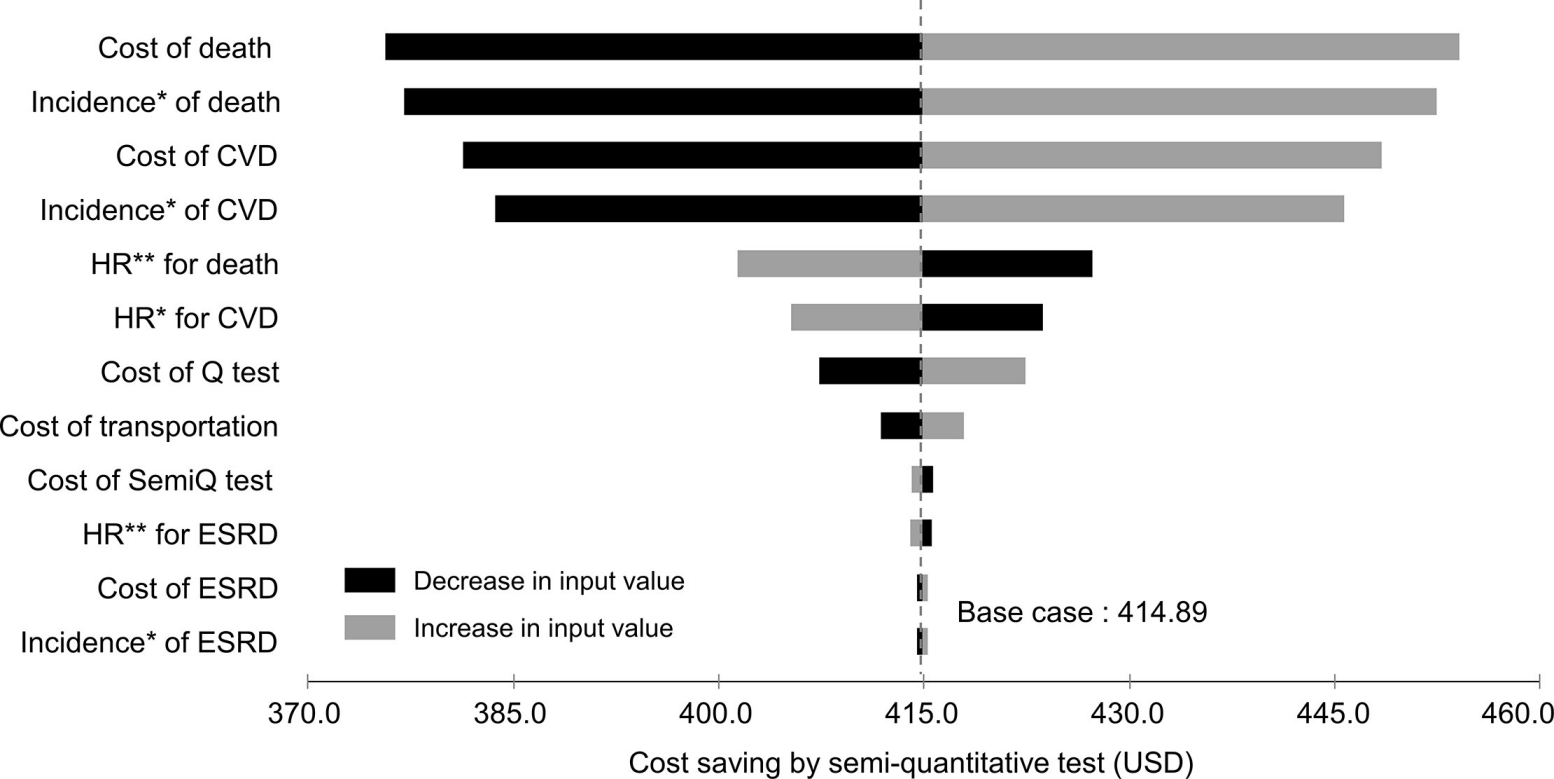
Tornado diagram of one-way sensitivity analyses. The vertical dashed line indicates the value in the base case analysis. Black bars represent the deviation resulting from the increase in corresponding input values. Gray bars represent the deviation resulting from the decrease in corresponding input values. * Incidences of clinical outcomes were converted to annual probabilities in the Markov model. ** Hazard ratios for clinical outcomes among diabetic patients (micro-or overt albuminuria versus normo-albuminuria). The values were used to calculate incidences of clinical outcomes in diabetic patients with micro- or overt albuminuria. Abbreviations: CVD, cardiovascular disease; ESRD, end-stage renal disease; HR, hazard ratio; Q test, quantitative test; SemiQ test, semi-quantitative test.

In the one-way sensitivity analysis, the amount of cost savings by the semi-quantitative method was changed according to a variety of input parameters. Although the cost of death changed with the broadest range (375.6 to 454.1 USD) depending on an input variation, the semi-quantitative method still saved costs. The result was depicted using a tornado plot (Fig 3).

### Validation of diagnostic accuracy of the semi-quantitative method and cost-saving analysis

The sensitivity and specificity were 83.8% (93/111) and 66.9% (214/320), respectively (S4 Table). The under-detected fraction was 4.2% (18/431) in the overall diabetic group. For the patients with eGFRs ≥60 ml/min/1.73 m^2^ and negative dipstick results, the sensitivity and specificity were 82.6% (38/46) and 73.3% (187/255), respectively. The under-detected fraction accounted for 2.7% (8/301) of the patients. To validate the cost-saving effect of the semi-quantitative method, we adjusted all fractions of the result to the Markov model. Finally, the semi-quantitative method saved 16.7% (339.6 USD) of costs compared to the quantitative test in one diabetic patient for 10 years (S5 Table).

In the one-way sensitivity analysis, the amount of cost savings yielded by the semi-quantitative method exhibited lowest and highest savings ranging from 0.4 to 54.5 USD according to the change in input parameters. Similarly to that in the development cohort, the cost of death showed the widest range in saving costs: 294.0 to 348.5 USD (S4 Fig).

## Discussion

Given that screening for albuminuria is recommended as an annual procedure for all diabetic patients, who comprise approximately 10% of the general population,[21] it is necessary to evaluate whether the semi-quantitative method, which is less expensive and more accessible, can replace the standard method.[5, 6, 22] In the present study, the semi-quantitative method showed comparable diagnostic accuracy to that identified in other published articles that evaluated albuminuria in diabetic patients.[23–27] Several reports have considered ethnicity, co-morbidities, the duration of diabetes, and the level of hemoglobin A1C.[25, 27] However, very few studies have focused on the clinical appropriateness of the test with respect to its cost effectiveness. This study focused on patients with normal renal function who had normal results on the dipstick test and revealed that the semi-quantitative method can be used as the standard screening tool for albuminuria in a specific subset of diabetic patients, with worthwhile economic benefit.

As expected, in almost 20% of the patients, albuminuria was not detected by the dipstick test even when the patients had uACR ≥30 mg/g. The semi-quantitative method identified albuminuria in over 80% of the above missed patients. The sensitivity was understandably lower in diabetic patients with normal renal function who were negative for proteinuria on the dipstick test. This lower sensitivity may have resulted from a lower prevalence of albuminuria in the study group than in the overall diabetic population. Despite relatively stricter criteria for inclusion, these results showed better sensitivity than those previously reported.[23, 24, 27]

Although only 3.2% of patients were under-detected in the study group, we carefully considered these results and adjusted the medical costs. We tried to calculate the cost-saving effectiveness with the highest possible estimate for the false negative fractions by using a more conservative approach, as mentioned above, because the patients logically could have experienced critical clinical outcomes for a year. CVD, ESRD, and mortality are the critical complications for patients with DN. Albuminuria is a good indicator and a well-known risk factor for predicting these complications and the long-term prognosis in DN patients.[14, 27–34] To reflect these risks in the cost, we selected matched articles by a systematic literature review. We selected the model closest to our study with information on kidney function and albuminuria, and predicted the relative risk by the sum of the HRs.

We contemplated the risk of clinical outcomes in diabetic patients with normal renal function and normo-albuminuria as a reference. The relative HR for mortality in DN patients with albuminuria compared to normo-albuminuric diabetic patients ranged from 1.02 to 2.15.[28, 30, 34–40] Only 2 of the 9 studies were completely matched and considered eGFR and albuminuria, and they showed results analogous to the others.[30, 40] Although several reports suggested a relative HR without considering the eGFR range, we included all the studies to obtain a more accurate result.[34, 36, 37] This approach could have ultimately caused an over-estimation of the costs for the false negative fraction.

There were 4 articles that reported the relative risk of CVD and ESRD together.[14, 35, 40, 41] The HRs for CVD were similar to those for all-cause mortality (1.27–1.89), but the HRs for ESRD were significantly higher (2.71–4.13) than those for the other outcomes. These results reflected the fact that albuminuria is involved in the development of ESRD. The annual progression rate from albuminuria to ESRD was only 0.1–0.3%.[42] However, most researchers did not show 1-year outcomes, so it was difficult to estimate the 1-year risk of the progression of disease. Therefore, we focused instead on the difference in the risk of albuminuria based on a normal eGFR. The pulled HRs for the clinical outcomes obtained from the reviewed studies were predisposed to overestimate the risk, which led to an increased cost for the false negative fraction.

The induced costs were inferred by multiplying the baseline medical cost by the relative hazard ratio for each clinical outcome in diabetic patients with albuminuria. The baseline medical costs were similar to those previously reported for each clinical outcome. For the CVD events, the event-year costs were reported as 56,445, 42,119, and 23,758 USD PPPY for myocardial infarction, stroke, and congestive heart failure, respectively, but the state costs reflecting annual resources were 1,904, 15,541, and 1,904 USD PPPY, respectively, for each outcome.[43] More recent research reported that the increased cost for overall CVD events in diabetic patients was 331.68 USD per person per month. Medicare costs attributable to CKD gradually increase as the stage increases; the adjusted annual cost for CKD stage IV progressing to ESRD was 43,272 USD.[44, 45] To compensate for this diversity and uncertainty for the induced costs, we performed a sensitivity analysis with various ranges in input parameters; particularly, for HRs, we used CIs in the meta-analysis. Moreover, the diversity of medical expenses necessarily reflects the socioeconomic status of the country and other circumstances. Thus, we calculated the induced costs with a more conservative approach, reflecting Korea’s socioeconomic status. To the best of our knowledge, this is the first study to consider the cost-saving effect of using the semi-quantitative method to detect albuminuria by calculating all the possibilities of disease progression based on the diagnostic accuracy. Notably, we considered the annual progression probability of critical clinical outcomes when the semi-quantitative method failed to detect albuminuria. Moreover, we extended the time horizon to 10 years in the Markov model and maximally assessed the potential health burden for the semi-quantitative method. To contemplate the probability of disease progression in patients with albuminuria, HRs were reassessed by evaluating the difference between patients with and without albuminuria. Despite adjusting the assessed HRs for each clinical outcome to the cost of the false negative fraction, the 10-year expected summed cost using quantitative testing was as high as 439.4 USD for one diabetic patient. In addition to the convenience of the semi-quantitative method, these results demonstrate how valuable this tool is for screening.

This study had several limitations. First, the proportion of patients with albuminuria was relatively small, which could have decreased the specificity of the semi-quantitative method. Second, the designs of the studies included in the pooled meta-analysis did not completely match our design; in particular, most of them did not consider the annual risk of disease. These unmatched criteria increased the medical costs in instances of false negative results. Third, although we performed a cost-saving analysis to elicit the economic value of the semi-quantitative method, it was limited to adopting a conservative methodological approach, such as a cost-effectiveness analysis (S2 File). However, we did not assess the cost by direct comparison but assessed it by considering the major health burden that could occur in diabetic patients with or without microalbuminuria over a 10-year time horizon. Thus, this approach to cost analysis could be considered as close to the conservative methodology. Based on these limitations, we should consider the possibility of an overestimation of the total cost and a diminution of the cost-saving effect of the semi-quantitative method.

In conclusion, the semi-quantitative method is eligible to serve as a screening tool for detecting albuminuria in diabetic patients. Furthermore, the use of this protocol was found to reduce the direct and indirect medical costs by >60% and provide excellent diagnostic accuracy. This approach can minimize time investment and patient inconvenience and improve the implementation of screening tests for albuminuria in diabetic patients.

## Supporting information

S1 Table Baseline characteristics of the development cohort. (DOCX)

S2 Table Baseline characteristics of the validation cohort. (DOCX)

S3 Table Reproducibility of the semi-quantitative assay for detection of microalbuminuria. (DOCX)

S4 Table The concordance of urine albumin/creatinine ratio using the semi-quantitative method and quantitative testing in the validation cohort. (DOCX)

S5 Table Cost-saving analysis of the semi-quantitative method compared with the quantitative method utilizing data from the validation cohort. (DOCX)

S1 Fig Differentiated groups according to the eGFR and urine albumin creatinine ratio criteria in the development cohort. Abbreviations: eGFR, estimated glomerular filtration rate; n, number; uACR, urine albumin-creatinine ratio.
(DOCX)

S2 Fig Within-run precision for urinary (A) albumin and (B) creatinine in the semi-quantitative method. The linearity was 0.853, 0.814, and 0.811 for urine albumin, and 0.931, 0.929, and 0.930 for urine creatinine at three different sites.
(DOCX)

S3 Fig Flow chart of studies through the systematic literature review. Abbreviations: eGFR, estimated glomerular filtration rate; n, number.
(DOCX)

S4 Fig Tornado diagram of one-way sensitivity analyses for the validation cohort. The vertical dashed line indicates the value in the base case analysis. Black bars represent the deviation resulting from the increase in corresponding input values. Gray bars represent the deviation resulting from the decrease in corresponding input values.
* Incidences of clinical outcomes were converted to annual probabilities in the Markov model.
** Hazard ratios for clinical outcomes among diabetic patients (micro- or overt albuminuria versus normo-albuminuria). The values were used to calculate incidences of clinical outcomes in diabetic patients with micro- or overt albuminuria.
Abbreviations: CVD, cardiovascular disease; ESRD, end-stage renal disease; HR, hazard ratio; Q test, quantitative test; SemiQ test, semi-quantitative test.
(DOCX)

S1 File PRISMA 2009 checklist. (PDF)

S2 File CHEERS checklist. (PDF)

## References

[1] Jin DC, Yun SR, Lee SW, Han SW, Kim W, Park J, et al Current characteristics of dialysis therapy in Korea: 2016 registry data focusing on diabetic patients. Kidney Res Clin Pract. 2018;37(1):20–9. 10.23876/j.krcp.2018.37.1.20 29629274 PMC5875573

[2] Haffner SM, Lehto S, Ronnemaa T, Pyorala K, Laakso M. Mortality from coronary heart disease in subjects with type 2 diabetes and in nondiabetic subjects with and without prior myocardial infarction. N Engl J Med. 1998;339(4):229–34. 10.1056/NEJM199807233390404 9673301

[3] de Boer IH, Rue TC, Hall YN, Heagerty PJ, Weiss NS, Himmelfarb J. Temporal trends in the prevalence of diabetic kidney disease in the United States. Jama. 2011;305(24):2532–9. 10.1001/jama.2011.861 21693741 PMC3731378

[4] Brancati FL, Whelton PK, Randall BL, Neaton JD, Stamler J, Klag MJ. Risk of end-stage renal disease in diabetes mellitus: a prospective cohort study of men screened for MRFIT. Multiple Risk Factor Intervention Trial. Jama. 1997;278(23):2069–74. 9403420

[5] Standards of medical care in diabetes—2013. Diabetes Care. 2013;36 Suppl 1:S11–66. 23264422 10.2337/dc13-S011 PMC3537269

[6] Zaharie F. K/DOQI clinical practice guidelines for chronic kidney disease: evaluation, classification, and stratification. Am J Kidney Dis. 2002;39(2 Suppl 1):S1–266. 11904577

[7] Price CP. Point of care testing. Bmj. 2001;322(7297):1285–8. 10.1136/bmj.322.7297.1285 11375233 PMC1120384

[8] Nichols JH, Christenson RH, Clarke W, Gronowski A, Hammett-Stabler CA, Jacobs E, et al Executive summary. The National Academy of Clinical Biochemistry Laboratory Medicine Practice Guideline: evidence-based practice for point-of-care testing. Clin Chim Acta. 2007;379(1–2):14–28; discussion 9–30. 10.1016/j.cca.2006.12.025 17270169

[9] Gialamas A, St John A, Laurence CO, Bubner TK. Point-of-care testing for patients with diabetes, hyperlipidaemia or coagulation disorders in the general practice setting: a systematic review. Fam Pract. 2010;27(1):17–24. 10.1093/fampra/cmp084 19969524

[10] Laurence CO, Moss JR, Briggs NE, Beilby JJ. The cost-effectiveness of point of care testing in a general practice setting: results from a randomised controlled trial. BMC Health Serv Res. 2010;10:165 10.1186/1472-6963-10-165 20546629 PMC2905350

[11] 2. Classification and Diagnosis of Diabetes: Standards of Medical Care in Diabetes-2019. Diabetes Care. 2019;42(Suppl 1):S13–s28. 10.2337/dc19-S002 30559228

[12] Tholen DW, Kallner A, Kennedy JW, Krouwer JS, Meier K. Evaluation of precision performance of quantitative measurement methods; approved guideline—second edition. Evaluation. 2004;24(25).

[13] Yokoyama H, Araki S, Haneda M, Matsushima M, Kawai K, Hirao K, et al Chronic kidney disease categories and renal-cardiovascular outcomes in type 2 diabetes without prevalent cardiovascular disease: a prospective cohort study (JDDM25). Diabetologia. 2012;55(7):1911–8. 10.1007/s00125-012-2536-y 22476921

[14] Ninomiya T, Perkovic V, de Galan BE, Zoungas S, Pillai A, Jardine M, et al Albuminuria and kidney function independently predict cardiovascular and renal outcomes in diabetes. J Am Soc Nephrol. 2009;20(8):1813–21. 10.1681/ASN.2008121270 19443635 PMC2723977

[15] Lee T, Biddle AK, Lionaki S, Derebail VK, Barbour SJ, Tannous S, et al Personalized prophylactic anticoagulation decision analysis in patients with membranous nephropathy. Kidney Int. 2014;85(6):1412–20. 10.1038/ki.2013.476 24336031 PMC4040154

[16] Lee J, Lee JS, Park SH, Shin SA, Kim K. Cohort Profile: The National Health Insurance Service-National Sample Cohort (NHIS-NSC), South Korea. Int J Epidemiol. 2017;46(2):e15 10.1093/ije/dyv319 26822938

[17] Olson M, Bailey MJ. Positive time preference. Journal of Political Economy. 1981;89(1):1–25.

[18] Ahn JH, Kim YH, Shin SJ, JY P. Cost of Medical Decision Making—Asian Joint Research on Effectiveness. National Evidence-based Healthcare Collaborating Agency; 2012.

[19] Kwon S, Lee T-j, Kim C-y. Republic of Korea health system review. Asia Pac. Observatory Public Health Syst. Policies. 2015;5(4).

[20] Cheol Seong S, Kim YY, Khang YH, Heon Park J, Kang HJ, Lee H, et al Data Resource Profile: The National Health Information Database of the National Health Insurance Service in South Korea. Int J Epidemiol. 2017;46(3):799–800. 10.1093/ije/dyw253 27794523 PMC5837262

[21] Organization WH. Global report on diabetes: World Health Organization; 2016 10.2337/db15-0956

[22] (NCD-RisC) NRFC. Worldwide trends in diabetes since 1980: a pooled analysis of 751 population-based studies with 4.4 million participants. Lancet. 2016;387(10027):1513–30. 10.1016/S0140-6736(16)00618-8 27061677 PMC5081106

[23] Le Floch JP, Marre M, Rodier M, Passa P. Interest of Clinitek Microalbumin in screening for microalbuminuria: results of a multicentre study in 302 diabetic patients. Diabetes Metab. 2001;27(1):36–9. 11240444

[24] Croal BL, Mutch WJ, Clark BM, Dickie A, Church J, Noble D, et al The clinical application of a urine albumin:creatinine ratio point-of-care device. Clin Chim Acta. 2001;307(1–2):15–21. 10.1016/s0009-8981(01)00450-8 11369331

[25] Meinhardt U, Ammann RA, Fluck C, Diem P, Mullis PE. Microalbuminuria in diabetes mellitus: efficacy of a new screening method in comparison with timed overnight urine collection. J Diabetes Complications. 2003;17(5):254–7. 10.1016/s1056-8727(02)00180-0 12954153

[26] Graziani MS, Gambaro G, Mantovani L, Sorio A, Yabarek T, Abaterusso C, et al Diagnostic accuracy of a reagent strip for assessing urinary albumin excretion in the general population. Nephrol Dial Transplant. 2009;24(5):1490–4. 10.1093/ndt/gfn639 19037085

[27] McTaggart MP, Price CP, Pinnock RG, Stevens PE, Newall RG, Lamb EJ. The diagnostic accuracy of a urine albumin-creatinine ratio point-of-care test for detection of albuminuria in primary care. Am J Kidney Dis. 2012;60(5):787–94. 10.1053/j.ajkd.2012.05.009 22721931

[28] Gerstein HC, Mann JF, Yi Q, Zinman B, Dinneen SF, Hoogwerf B, et al Albuminuria and risk of cardiovascular events, death, and heart failure in diabetic and nondiabetic individuals. Jama. 2001;286(4):421–6. 10.1001/jama.286.4.421 11466120

[29] Valmadrid CT, Klein R, Moss SE, Klein BE. The risk of cardiovascular disease mortality associated with microalbuminuria and gross proteinuria in persons with older-onset diabetes mellitus. Arch Intern Med. 2000;160(8):1093–100. 10.1001/archinte.160.8.1093 10789601

[30] O'Hare AM, Hailpern SM, Pavkov ME, Rios-Burrows N, Gupta I, Maynard C, et al Prognostic implications of the urinary albumin to creatinine ratio in veterans of different ages with diabetes. Arch Intern Med. 2010;170(11):930–6. 10.1001/archinternmed.2010.129 20548004

[31] Yuyun MF, Khaw KT, Luben R, Welch A, Bingham S, Day NE, et al Microalbuminuria independently predicts all-cause and cardiovascular mortality in a British population: The European Prospective Investigation into Cancer in Norfolk (EPIC-Norfolk) population study. Int J Epidemiol. 2004;33(1):189–98. 10.1093/ije/dyh008 15075168

[32] Klausen K, Borch-Johnsen K, Feldt-Rasmussen B, Jensen G, Clausen P, Scharling H, et al Very low levels of microalbuminuria are associated with increased risk of coronary heart disease and death independently of renal function, hypertension, and diabetes. Circulation. 2004;110(1):32–5. 10.1161/01.CIR.0000133312.96477.48 15210602

[33] Berhane AM, Weil EJ, Knowler WC, Nelson RG, Hanson RL. Albuminuria and estimated glomerular filtration rate as predictors of diabetic end-stage renal disease and death. Clin J Am Soc Nephrol. 2011;6(10):2444–51. 10.2215/CJN.00580111 21852671 PMC3186453

[34] Ritte R, Luke J, Nelson C, Brown A, O'Dea K, Jenkins A, et al Clinical outcomes associated with albuminuria in central Australia: a cohort study. BMC Nephrol. 2016;17(1):113 10.1186/s12882-016-0328-1 27495237 PMC4974695

[35] Tong PC, Kong AP, So WY, Yang X, Ng MC, Ho CS, et al Interactive effect of retinopathy and macroalbuminuria on all-cause mortality, cardiovascular and renal end points in Chinese patients with Type 2 diabetes mellitus. Diabet Med. 2007;24(7):741–6. 10.1111/j.1464-5491.2007.02145.x 17403120

[36] Targher G, Zoppini G, Chonchol M, Negri C, Stoico V, Perrone F, et al Glomerular filtration rate, albuminuria and risk of cardiovascular and all-cause mortality in type 2 diabetic individuals. Nutr Metab Cardiovasc Dis. 2011;21(4):294–301. 10.1016/j.numecd.2009.10.002 20096544

[37] Konta T, Kudo K, Sato H, Ichikawa K, Ikeda A, Suzuki K, et al Albuminuria is an independent predictor of all-cause and cardiovascular mortality in the Japanese population: the Takahata study. Clin Exp Nephrol. 2013;17(6):805–10. 10.1007/s10157-013-0770-3 23345069

[38] Svensson MK, Cederholm J, Eliasson B, Zethelius B, Gudbjornsdottir S. Albuminuria and renal function as predictors of cardiovascular events and mortality in a general population of patients with type 2 diabetes: a nationwide observational study from the Swedish National Diabetes Register. Diab Vasc Dis Res. 2013;10(6):520–9. 10.1177/1479164113500798 24002670

[39] Hsieh YT, Kuo JF, Su SL, Chen JF, Chen HC, Hsieh MC. Subnormal Estimated Glomerular Filtration Rate Strongly Predict Incident Cardiovascular Events in Type 2 Diabetic Chinese Population With Normoalbuminuria. Medicine (Baltimore). 2016;95(2):e2200. 26765399 10.1097/MD.0000000000002200 PMC4718225

[40] Wada T, Haneda M, Furuichi K, Babazono T, Yokoyama H, Iseki K, et al Clinical impact of albuminuria and glomerular filtration rate on renal and cardiovascular events, and all-cause mortality in Japanese patients with type 2 diabetes. Clin Exp Nephrol. 2014;18(4):613–20. 10.1007/s10157-013-0879-4 24132561

[41] So WY, Kong AP, Ma RC, Ozaki R, Szeto CC, Chan NN, et al Glomerular filtration rate, cardiorenal end points, and all-cause mortality in type 2 diabetic patients. Diabetes Care. 2006;29(9):2046–52. 10.2337/dc06-0248 16936151

[42] Adler AI, Stevens RJ, Manley SE, Bilous RW, Cull CA, Holman RR. Development and progression of nephropathy in type 2 diabetes: the United Kingdom Prospective Diabetes Study (UKPDS 64). Kidney Int. 2003;63(1):225–32. 10.1046/j.1523-1755.2003.00712.x 12472787

[43] Ward A, Alvarez P, Vo L, Martin S. Direct medical costs of complications of diabetes in the United States: estimates for event-year and annual state costs (USD 2012). J Med Econ. 2014;17(3):176–83. 10.3111/13696998.2014.882843 24410011

[44] Vupputuri S, Kimes TM, Calloway MO, Christian JB, Bruhn D, Martin AA, et al The economic burden of progressive chronic kidney disease among patients with type 2 diabetes. J Diabetes Complications. 2014;28(1):10–6. 10.1016/j.jdiacomp.2013.09.014 24211091

[45] Honeycutt AA, Segel JE, Zhuo X, Hoerger TJ, Imai K, Williams D. Medical costs of CKD in the Medicare population. J Am Soc Nephrol. 2013;24(9):1478–83. 10.1681/ASN.2012040392 23907508 PMC3752941

