# Supplementary material for: Can a semi-quantitative method replace the current quantitative method for the annual screening of microalbuminuria in patients with diabetes? Diagnostic accuracy and cost-saving analysis considering the potential health burden
Source: PLoS One. 2020 Jan 21;15(1):e0227694. doi: 10.1371/journal.pone.0227694 (PMC6974274; doi:10.1371/journal.pone.0227694)
Supplement: S1 Table — (DOCX) [file pone.0227694.s001.docx]

**S1 Table.** Baseline characteristics of the development cohort

|  | All diabetes  (n = 1,881) | Diabetes with eGFR ≥60 ml/min/m^2^ and dipstick (-)  (n = 1,110) |
| --- | --- | --- |
| age, year [ ]^*^ | 66.0 [58.0–73.0] | 64.0 [57.0–71.0] |
| <40, n (%) | 83 (4.4) | 53 (4.8) |
| 40–55, n (%) | 252 (13.4) | 150 (13.5) |
| 55–70, n (%) | 828 (44.0) | 549 (49.5) |
| ≥70, n (%) | 718 (38.2) | 358 (32.3) |
| male, n (%) | 1043 (55.4) | 576 (51.9) |
| WBC, x10^3^/ul | 7.0 ± 2.4 | 6.8 ± 2.0 |
| hemoglobin, g/dL | 13.4 ± 1.9 | 13.8 ± 1.6 |
| platelet, x10^3^/ul | 231.8 ± 69.5 | 233.8 ± 65.6 |
| calcium, mg/dL | 9.3 ± 0.5 | 9.4 ± 0.4 |
| phosphorus, mg/dL | 3.6 ± 0.6 | 3.5 ± 0.5 |
| glucose, mg/dL | 139.8 ± 42.4 | 139.1 ± 38.0 |
| uric acid, mg/dL | 5.5 ± 1.6 | 5.1 ± 1.3 |
| cholesterol, mg/dL | 162.3 ± 37.2 | 162.7 ± 34.1 |
| albumin, g/dL | 4.3 ± 0.4 | 4.3 ± 0.3 |
| eGFR, CKD-EPI, ml/min/m^2^ | 74.4 ± 26.7 | 88.0 ± 16.1 |
| uACR, mg/g Cr [ ]^*^ | 22.0 [10.0–108.5] | 12.0 [8.0–23.0] |
| <30, n (%) | 1071 (57.5) | 917 (82.6) |
| 30–300, n (%) | 480 (25.5) | 186 (16.8) |
| ≥300, n (%) | 320 (17.0) | 7 (0.6) |

*, Value was demonstrated by median [inter quartile range]

eGFR, estimated glomerular filtration rate; CKD-EPI, Chronic Kidney Disease Epidemiology Collaboration; uACR, urine albumin to creatinine ratio
