# Supplementary material for: Can a semi-quantitative method replace the current quantitative method for the annual screening of microalbuminuria in patients with diabetes? Diagnostic accuracy and cost-saving analysis considering the potential health burden
Source: PLoS One. 2020 Jan 21;15(1):e0227694. doi: 10.1371/journal.pone.0227694 (PMC6974274; doi:10.1371/journal.pone.0227694)
Supplement: S2 Table — (DOCX) [file pone.0227694.s002.docx]

**S2 Table.** Baseline characteristics of the validation cohort

|  | All diabetes  (n = 431) | Diabetes with eGFR ≥60 ml/min/m^2^ and dipstick (-)  (n = 301) |
| --- | --- | --- |
| age, year [ ]^*^ | 63.0 [53.0–70.0] | 62.0 [52.0–69.0] |
| <40, n (%) | 36 (8.4) | 28 (9.3) |
| 40–55, n (%) | 87 (20.2) | 64 (21.3) |
| 55–70, n (%) | 196 (45.5) | 146 (48.5) |
| ≥70, n (%) | 112 (26.0) | 63 (20.9) |
| male, n (%) | 216 (50.1) | 151 (50.2) |
| WBC, x10^3^/ul | 7.0 ± 2.1 | 6.8 ± 2.1 |
| hemoglobin, g/dL | 13.1 ± 2.0 | 13.3 ± 1.9 |
| platelet, x10^3^/ul | 245.0 ± 76.6 | 248.5 ± 76.7 |
| calcium, mg/dL | 9.4 ± 0.6 | 9.4 ± 0.5 |
| phosphorus, mg/dL | 3.6 ± 0.7 | 3.6 ± 0.6 |
| glucose, mg/dL | 149.5 ± 65.0 | 143.5 ±56.8 |
| uric acid, mg/dL | 5.1 ± 1.6 | 4.8 ± 1.5 |
| cholesterol, mg/dL | 154.4 ± 41.4 | 155.6 ± 40.8 |
| albumin, g/dL | 4.4 ± 0.7 | 4.4 ± 0.7 |
| eGFR, CKD-EPI, ml/min/m^2^ | 85.3 ± 23.0 | 92.2 ± 16.4 |
| uACR, mg/g Cr [ ]^*^ | 11.0 [5.0-32.0] | 8.0 [4.0-18.0] |
| <30, n (%) | 320 (74.2) | 255 (84.7) |
| 30–300, n (%) | 96 (22.3) | 43 (14.3) |
| ≥300, n (%) | 15 (3.5) | 3 (1.0) |

*, Value was demonstrated by median [inter quartile range]

eGFR, estimated glomerular filtration rate; CKD-EPI, Chronic Kidney Disease Epidemiology Collaboration; uACR, urine albumin to creatinine ratio
