# Supplementary material for: Can a semi-quantitative method replace the current quantitative method for the annual screening of microalbuminuria in patients with diabetes? Diagnostic accuracy and cost-saving analysis considering the potential health burden
Source: PLoS One. 2020 Jan 21;15(1):e0227694. doi: 10.1371/journal.pone.0227694 (PMC6974274; doi:10.1371/journal.pone.0227694)
Supplement: S3 Table — (DOCX) [file pone.0227694.s003.docx]

**S3 Table.** Reproducibility of the semi-quantitative assay for detection of microalbuminuria

| Albumin (10 days) | | | | | | | | | | | | | | | | | | | | | | | | | | |
| --- | --- | --- | --- | --- | --- | --- | --- | --- | --- | --- | --- | --- | --- | --- | --- | --- | --- | --- | --- | --- | --- | --- | --- | --- | --- | --- |
| Specimen | Site 1(100)^a^ | | | | | | Site 2(100)^a^ | | | | | | | Site 3(100)^a^ | | | | | | | Total(300)^b^ | | | | | |
|  | Agr. % | Ave. | STD | | CV% | | Agr. % | | Ave. | STD | | CV% | | Agr. % | | Ave. | STD | | CV% | | Agr. % | Ave. | STD | | CV% |  |
| 0.2 | 100% | 1.4 | 0.8 | | 57 | | 100% | | 1.8 | 0.4 | | 22 | | 100% | | 1.8 | 0.9 | | 49 | | 100% | 1.7 | 0.7 | | 44 |  |
| 30.2 | 100% | 18.2 | 1.5 | | 8 | | 100% | | 18.6 | 1.3 | | 7 | | 100% | | 18.6 | 1.2 | | 6 | | 100% | 18.5 | 1.3 | | 7 |  |
| 80.0 | 100% | 22.5 | 1.5 | | 7 | | 100% | | 21.6 | 0.8 | | 4 | | 100% | | 22.2 | 1.1 | | 5 | | 100% | 22.0 | 1.2 | | 5 |  |
| 150.3 | 100% | 31.2 | 2.4 | | 8 | | 100% | | 29.5 | 1.1 | | 4 | | 100% | | 30.2 | 1.1 | | 4 | | 100% | 30.3 | 1.7 | | 6 |  |
|  | | | | | | | | | | | | | | | | | | | | | | | | | | |
| Creatinine (10 days) | | | | | | | | | | | | | | | | | | | | | | | | | | |
| Specimen | Site 1(100)^a^ | | | | | | Site 2(100)^a^ | | | | | | | Site 3(100)^a^ | | | | | | | Total(300)^b^ | | | | | |
|  | Agr. % | Ave. | | STD | | CV% | | Agr. % | Ave. | | STD | | CV% | | Agr. % | Ave. | | STD | | CV% | Agr. % | Ave. | | STD | CV% |  |
| 10.1 | 100% | 7.4 | | 2.1 | | 28 | | 100% | 8.0 | | 1.8 | | 22 | | 100% | 7.1 | | 1.3 | | 19 | 100% | 7.1 | | 1.8 | 24 |  |
| 50.1 | 100% | 58.7 | | 2.5 | | 4 | | 100% | 58.8 | | 2.6 | | 4 | | 100% | 54.8 | | 1.7 | | 3 | 100% | 57.4 | | 2.9 | 5 |  |
| 100.6 | 100% | 85.4 | | 2.5 | | 3 | | 100% | 85.6 | | 3.1 | | 4 | | 100% | 82.5 | | 2.9 | | 4 | 100% | 84.5 | | 3.1 | 4 |  |
| 200.2 | 100% | 113.4 | | 2.4 | | 2 | | 100% | 111.4 | | 3.2 | | 3 | | 100% | 110.8 | | 3.5 | | 3 | 100% | 111.9 | | 3.4 | 3 |  |
| 300.2 | 100% | 134.9 | | 1.7 | | 1 | | 100% | 134.4 | | 3.2 | | 2 | | 100% | 134.3 | | 3.0 | | 2 | 100% | 133.7 | | 3.0 | 2 |  |

a: Test number at different sites, b: Test number of all sites

Agr, agreement; Ave, average; CV, coefficient variance; STD, standard deviation;
