# Supplementary material for: Can a semi-quantitative method replace the current quantitative method for the annual screening of microalbuminuria in patients with diabetes? Diagnostic accuracy and cost-saving analysis considering the potential health burden
Source: PLoS One. 2020 Jan 21;15(1):e0227694. doi: 10.1371/journal.pone.0227694 (PMC6974274; doi:10.1371/journal.pone.0227694)
Supplement: S4 Table — (DOCX) [file pone.0227694.s004.docx]

**S4 Table.** The concordance of urine albumin/creatinine ratio using the semi-quantitative method and the quantitative testing in the validation cohort

|  | Quantitative testing  (mg/g) | semi-quantitative method | | | Sensitivity | Specificity | *Over detected fraction | **Under detected fraction |
| --- | --- | --- | --- | --- | --- | --- | --- | --- |
|  |  | <30 | 30–300 | ≥300 |  |  |  |  |
| All diabetes  (n = 431) | <30 | 214 (66.9) | 102 (31.9) | 4 (1.2) | 83.8 | 66.9 | 24.6 | 4.2 |
|  | 30–300 | 18 (18.8) | 52 (54.2) | 26 (27.1) |  |  |  |  |
|  | ≥300 | 0 (0.0) | 1 (6.7) | 14 (93.3) |  |  |  |  |
| Diabetes with eGFR ≥60 ml/min/m^2^ and dipstick (-)  (n = 301) | <30 | 187 (73.3) | 64 (25.1) | 4 (1.6) | 82.6 | 73.3 | 22.6 | 2.7 |
|  | 30–300 | 8 (18.6) | 28 (65.1) | 7 (16.3) |  |  |  |  |
|  | ≥300 | 0 (0.0) | 1 (33.3) | 2 (66.7) |  |  |  |  |

*, number of false positive /total number of population x 100

**, number of false negative/total number of population x 100

eGFR, estimated glomerular filtration rate
