# Supplementary material for: Can a semi-quantitative method replace the current quantitative method for the annual screening of microalbuminuria in patients with diabetes? Diagnostic accuracy and cost-saving analysis considering the potential health burden
Source: PLoS One. 2020 Jan 21;15(1):e0227694. doi: 10.1371/journal.pone.0227694 (PMC6974274; doi:10.1371/journal.pone.0227694)
Supplement: S5 Table — (DOCX) [file pone.0227694.s005.docx]

**S5 Table.** Cost-saving analysis of the semi-quantitative compared with the quantitative method utilizing data from the validation cohort.

|  | |  | |  |  | | Quantitative strategy  (per person) | | | Semi-quantitative strategy  (per person) | |  |
| --- | --- | --- | --- | --- | --- | --- | --- | --- | --- | --- | --- | --- |
|  | Initial screening for urine albumin to creatinine ratio, $* | | | | | | | 86.89 | 4.63 | |  |  |
|  | Transportation, $ | | | | | | | 90.65 | 49.86 | |  |  |
|  |  | |  | | | *Total costs for screening, $* | | *177.54* | *54.48* | |  |  |
|  | Confirmatory quantitative test for positive fraction at semi-quantitative strategy, $ | | | | | | |  |  | |  |  |
|  |  | | Quantitative test | | | | | - | 33.66 | |  |  |
|  |  | | Transportation | | | | | - | 17.56 | |  |  |
|  | Cost of under detected fraction, $ (95% CI) | | | | | | |  |  | |  |  |
|  |  | | End-stage renal disease | | | | | 16.93 | 15.66 | |  |  |
|  |  | | Cardiovascular disease | | | | | 832.88 | 709.52 | |  |  |
|  |  | | All-cause mortality | | | | | 1004.56 | 861.48 | |  |  |
|  |  | |  | | | *Summed costs (crude), $* | | *2031.91* | *1692.36* | |  |  |
|  |  | |  | | | *Summed costs (discounted), $* | | *1864.47* | *1543.23* | |  |  |
|  |  | |  | | | *Saved cost (crude), $* | |  | *339.55* | |  |  |
|  |  | |  | | | *Saved cost (crude), %* | |  | *16.7* | |  |  |
| *1 USD = 1,160 KRW (Annual average exchange rate, 2016). Baseline year of cost, 2016. | | | | | | | | | | | | |
