# Supplementary material for: Can a semi-quantitative method replace the current quantitative method for the annual screening of microalbuminuria in patients with diabetes? Diagnostic accuracy and cost-saving analysis considering the potential health burden
Source: PLoS One. 2020 Jan 21;15(1):e0227694. doi: 10.1371/journal.pone.0227694 (PMC6974274; doi:10.1371/journal.pone.0227694)
Supplement: S1 Fig — Abbreviations: eGFR, estimated glomerular filtration rate; n, number; uACR, urine albumin-creatinine ratio. (DOCX) [file pone.0227694.s006.docx]

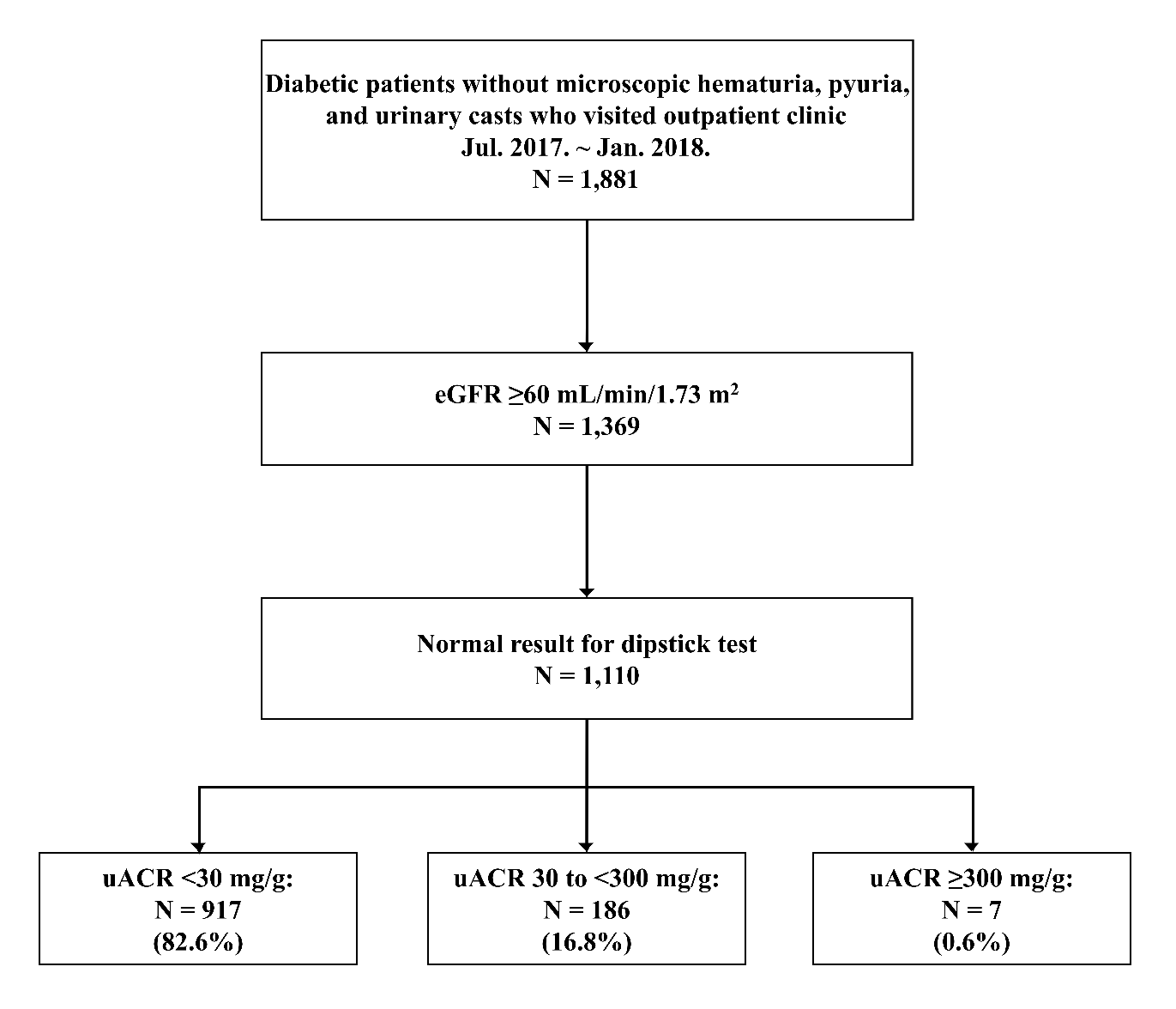
**S1 Fig**. Differentiated groups according to the eGFR and urine albumin creatinine ratio criteria in the development cohort

Abbreviations: eGFR, estimated glomerular filtration rate; n, number; uACR, urine albumin-creatinine ratio
