# Supplementary material for: Can a semi-quantitative method replace the current quantitative method for the annual screening of microalbuminuria in patients with diabetes? Diagnostic accuracy and cost-saving analysis considering the potential health burden
Source: PLoS One. 2020 Jan 21;15(1):e0227694. doi: 10.1371/journal.pone.0227694 (PMC6974274; doi:10.1371/journal.pone.0227694)
Supplement: S2 Fig — Within-run precision for urinary (A) albumin and (B) creatinine in the semi-quantitative method. The linearity was 0.853, 0.814, and 0.811 for urine albumin, and 0.931, 0.929, and 0.930 for urine creatinine at three different sites. (DOCX) [file pone.0227694.s007.docx]

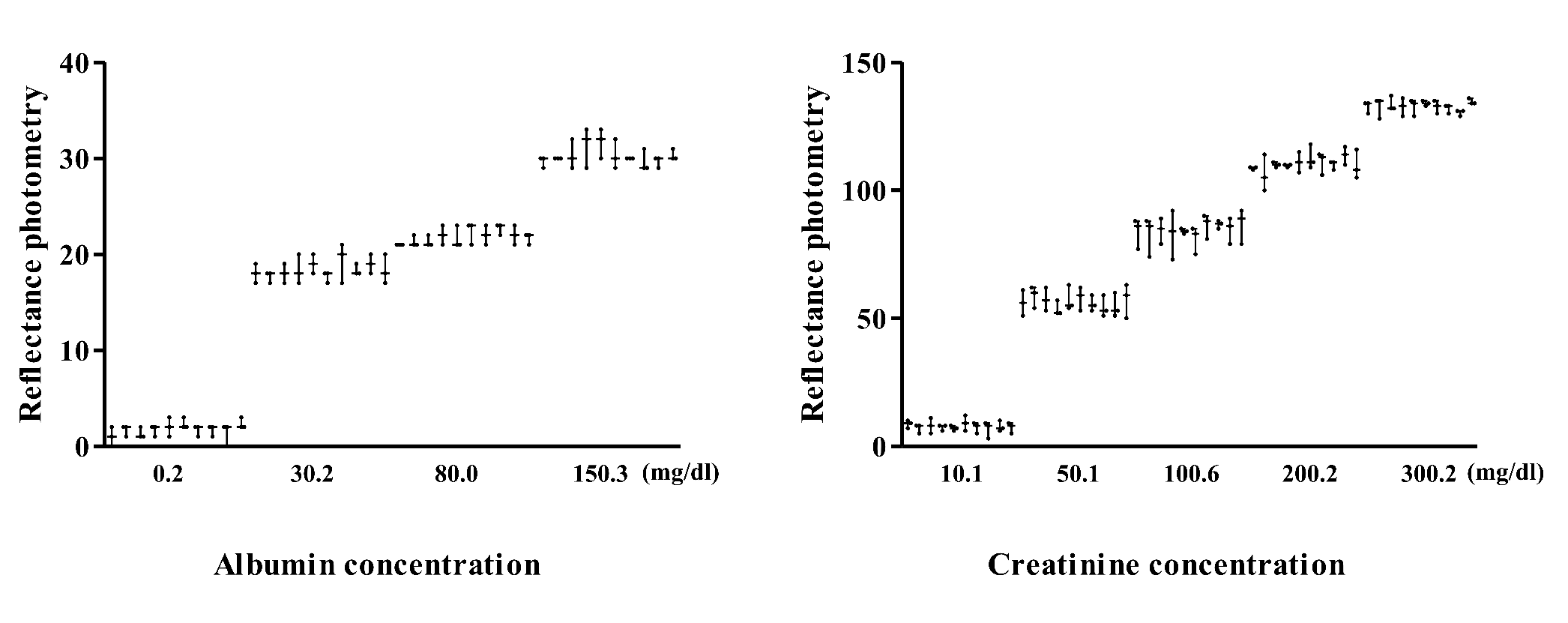


**S2 Fig**. Within-run precision for urinary (A) albumin and (B) creatinine in the semi-quantitative method.

The linearity was 0.853, 0.814, and 0.811 for urine albumin, and 0.931, 0.929, and 0.930 for urine creatinine at three different sites.
