# Supplementary material for: Can a semi-quantitative method replace the current quantitative method for the annual screening of microalbuminuria in patients with diabetes? Diagnostic accuracy and cost-saving analysis considering the potential health burden
Source: PLoS One. 2020 Jan 21;15(1):e0227694. doi: 10.1371/journal.pone.0227694 (PMC6974274; doi:10.1371/journal.pone.0227694)
Supplement: S3 Fig — Abbreviations: eGFR, estimated glomerular filtration rate; n, number. (DOCX) [file pone.0227694.s008.docx]

**
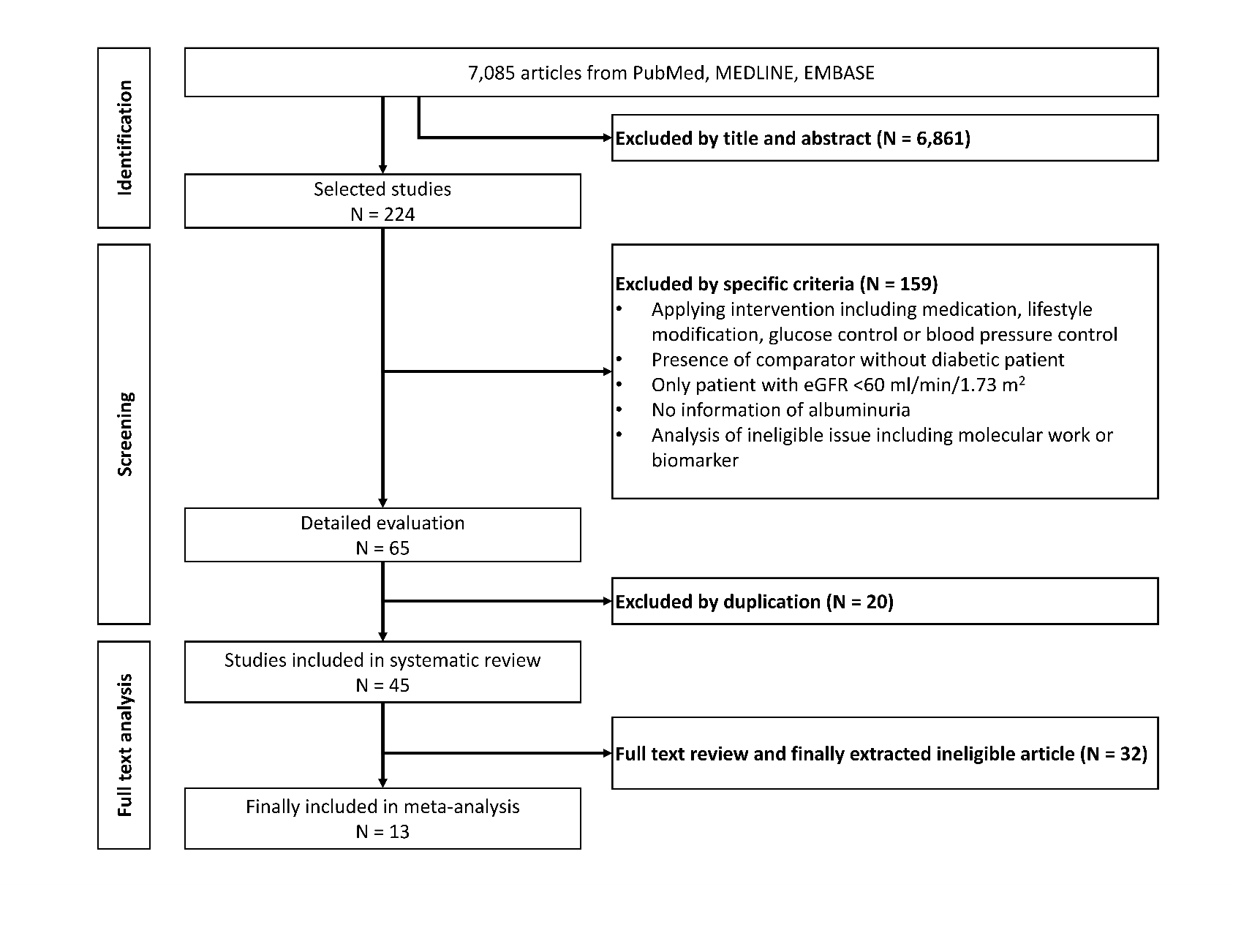
**

**S3 Fig**. Flow chart of studies through the systematic literature review

Abbreviations: eGFR, estimated glomerular filtration rate; n, number.
