# Supplementary material for: Can a semi-quantitative method replace the current quantitative method for the annual screening of microalbuminuria in patients with diabetes? Diagnostic accuracy and cost-saving analysis considering the potential health burden
Source: PLoS One. 2020 Jan 21;15(1):e0227694. doi: 10.1371/journal.pone.0227694 (PMC6974274; doi:10.1371/journal.pone.0227694)
Supplement: S4 Fig — The vertical dashed line indicates the value in the base case analysis. Black bars represent the deviation resulting from the increase in corresponding input values. Gray bars represent the deviation resulting from the decrease in corresponding input values. * Incidences of clinical outcomes were converted to annual probabilities in the Markov model. ** Hazard ratios for clinical outcomes among diabetic patients (micro- or overt albuminuria versus normo-albuminuria). The values were used to calculate incidences of clinical outcomes in diabetic patients with micro- or overt albuminuria. Abbreviations: CVD, cardiovascular disease; ESRD, end-stage renal disease; HR, hazard ratio; Q test, quantitative test; SemiQ test, semi-quantitative test. (DOCX) [file pone.0227694.s009.docx]

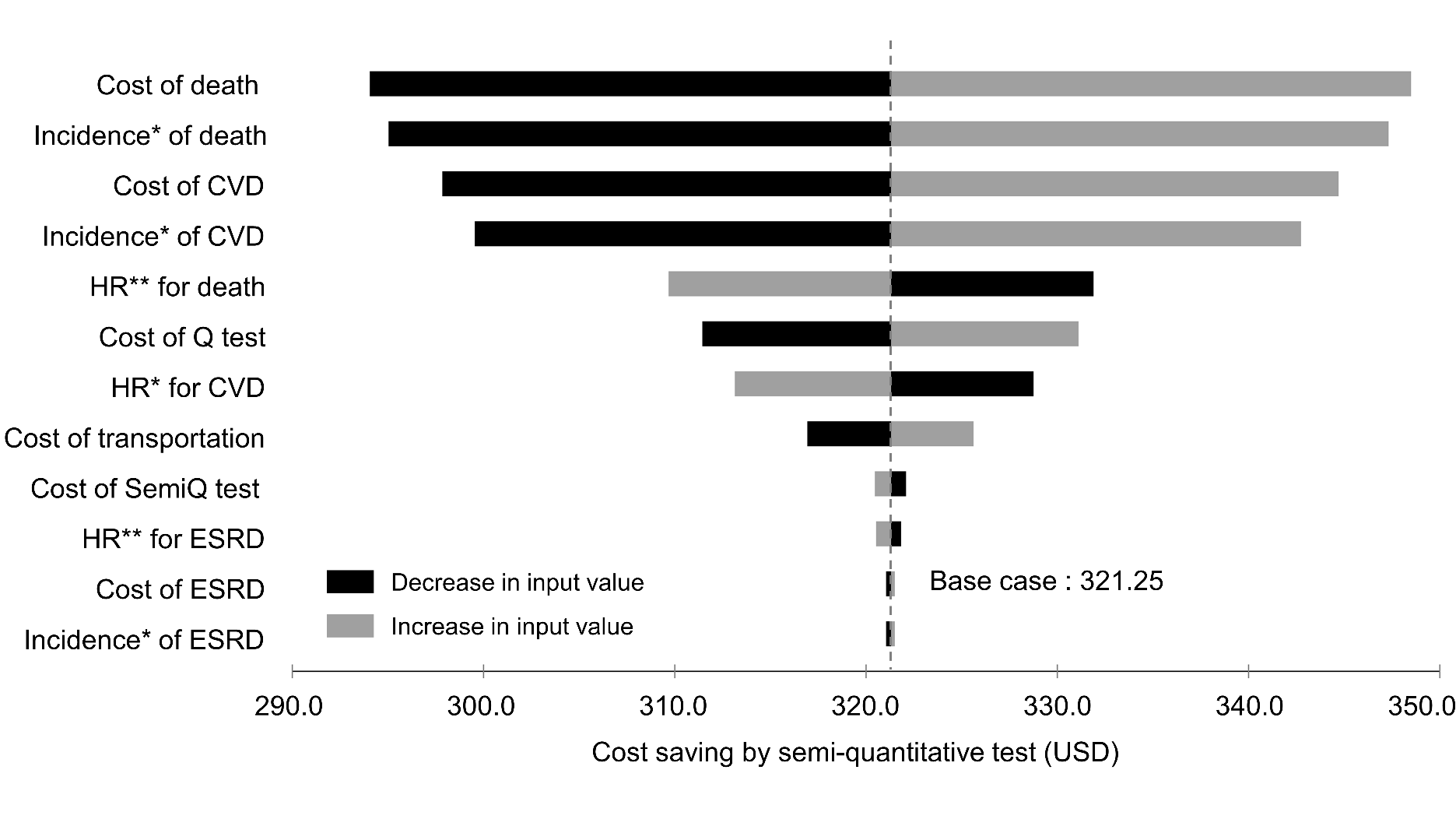


**S4 Fig**. Tornado diagram of one-way sensitivity analyses for validation cohort.

Vertical dashed line indicates the value in the base case analysis. Black bars represent the deviation resulting from the increase in corresponding input values. Grey bars represent the deviation resulting from the decrease in corresponding input values.
* Incidences of clinical outcomes were converted to annual probabilities in the Markov model.

** Hazard ratios for clinical outcomes among diabetic patients (micro-or overt albuminuria versus normo-albuminuria). The values were used to calculate incidences of clinical outcomes in diabetic patients with micro- or overt albuminuria.
Abbreviations: CVD, cardiovascular disease; ESRD, end-stage renal disease; HR, hazard ratio; Q test, quantitative test; SemiQ test, semi-quantitative test.
